# Strategies to Optimize Adult Stem Cell Therapy for Tissue Regeneration

**DOI:** 10.3390/ijms17060982

**Published:** 2016-06-21

**Authors:** Shan Liu, Jingli Zhou, Xuan Zhang, Yang Liu, Jin Chen, Bo Hu, Jinlin Song, Yuanyuan Zhang

**Affiliations:** 1 Chongqing Key Laboratory for Oral Diseases and Biomedical Sciences, Chongqing 401147, China; 15823106296@163.com (S.L.); journeyzone@163.com (J.Z.); 13677691923@163.com (X.Z.); 13657638643@163.com (Y.L.); ChenJindentistry@163.com (J.C.); huboisthebest@sina.com (B.H.); 2 College of Stomatology, Chongqing Medical University, Chongqing 401147, China; 3 Chongqing Municipal Key Laboratory of Oral Biomedical Engineering of Higher Education, Chongqing 401147, China; 4 Wake Forest Institute for Regenerative Medicine, Wake Forest School of Medicine, Medical Center Blvd., Winston-Salem, NC 27157, USA

**Keywords:** stem cells, stem cell therapy, optimizing strategy, tissue repair, tissue regeneration

## Abstract

Stem cell therapy aims to replace damaged or aged cells with healthy functioning cells in congenital defects, tissue injuries, autoimmune disorders, and neurogenic degenerative diseases. Among various types of stem cells, adult stem cells (*i.e.*, tissue-specific stem cells) commit to becoming the functional cells from their tissue of origin. These cells are the most commonly used in cell-based therapy since they do not confer risk of teratomas, do not require fetal stem cell maneuvers and thus are free of ethical concerns, and they confer low immunogenicity (even if allogenous). The goal of this review is to summarize the current state of the art and advances in using stem cell therapy for tissue repair in solid organs. Here we address key factors in cell preparation, such as the source of adult stem cells, optimal cell types for implantation (universal mesenchymal stem cells *vs.* tissue-specific stem cells, or induced *vs.* non-induced stem cells), early or late passages of stem cells, stem cells with endogenous or exogenous growth factors, preconditioning of stem cells (hypoxia, growth factors, or conditioned medium), using various controlled release systems to deliver growth factors with hydrogels or microspheres to provide apposite interactions of stem cells and their niche. We also review several approaches of cell delivery that affect the outcomes of cell therapy, including the appropriate routes of cell administration (systemic, intravenous, or intraperitoneal *vs.* local administration), timing for cell therapy (immediate *vs.* a few days after injury), single injection of a large number of cells *vs.* multiple smaller injections, a single site for injection *vs.* multiple sites and use of rodents *vs.* larger animal models. Future directions of stem cell-based therapies are also discussed to guide potential clinical applications.

## 1. Introduction

Currently, adult stem cells are the primary source of cells studied in tissue regeneration. These cells are most from hematopoietic, mesenchymal, epithelial and neural cell lineages. As the most commonly used source in cell therapy, mesenchymal stem cells (MSCs) possess high proliferation ability, paracrine effect, multipotent differentiation potential immunomodulatory capacity [1,2], and profiles of cell surface markers [3]. Furthermore, MSCs can home to tumors carrying with anticancer agents and have an important role in the resistance to various anti-cancer drugs. Table 1 summarizes key therapeutic properties of MSCs for use in tissue regeneration therapy. MSCs are potential in the treatment of various congenital defects (*i.e.*, congenital heart disease [4], congenital diaphragmatic hernia [5], myelomeningocele [6]), trauma or ischemia injuries (*i.e.*, myocardial ischemia [7], spinal injury [8], kidney failure [9]), immune disorders (diabetes [10], arthritis [11], multiple sclerosis [12]) and degenerated diseases (*i.e.*, intervertebral disc degeneration [13], muscle degeneration [14], sensorineural hearing loss [15]). However, a major difficulty with stem cell therapy is to maintain cell viability, properties and function of stem cell before and after implantation *in vivo*. Once stem cells are isolated from the native tissue environment, they quickly lose the niche and function they had when growing in culture dishes. In addition, they shorten cell lifespan because of over-expansion *in vitro*. Furthermore, cellular DNA tends to be instable during long-term culture. Such cells implanted in the host lead low rates of cell survival, and poor outcomes in-growth, homing, differentiation and paracrine effects. On the other hand, the environment of implanted site, such as blood supply, the collagen density of tissue (scaring tissue formation) and the numbers of existing endogenous stem cells, greatly affects the fate of grafted cells and the effect of cell-therapy. Clearly, longer the graft cells survive in the appropriate environments provided, the more trophic factors secreted, and better tissue regeneration induced. Thus, extension of transplanted cell life-span and stimulation of activity of paracrine effects can improve the tissue repair. Therefore, it is essential to optimize the strategies for stem cell therapy for tissue regeneration in solid organs. In this review, several general concepts and strategies have been addressed for cell therapy, which could be specifically justified when applied in different tissues or organs.

## 2. Sources of MSCs

MSCs were initially recognized in bone marrow and afterward revealed in many other organs and tissues. These adult stem cells appear to have a pericytic or fibroblastic origin from various tissue environments. Recently, more evidences have indicated that MSCs exist nearly in any vascularized tissues *in vivo* [16]. However, a large difference in their expression is noted in various sources of MSCs. While bone marrow [17] is the broadly identified source of adult stem cells, alternative sources of MSC-like cells has been gradually recognized, including adipose tissue [18], dental pulp [19], synovial membrane [20], periodontal ligament [21], hair follicle [22], endometrium [23], placenta [24], umbilical cord [25], peripheral blood [26], umbilical cord blood [27], amniotic fluid [28], menstrual blood [29], milk [30] and urine [31]. Although the precise identity of these stem cells *in vivo* is not well defined, a number of surface antigens, instead of a single molecule, have been widely used in characterization of MSCs *in vitro*. These surface markers may be associated to their stemness, such as expression of CD10, CD13, CD29, CD44, CD73, CD90, CD105, CD146, CD271, Stro-1 and SSEA-4, low/negative expression of CD14 (CD11b), CD19 (or CD79a), CD34, CD45 and HLA-DR [32,33,34,35,36,37,38].

Among different types of MSCs, they possess similar multipotential differentiation potential although their differentiation efficacy varies during specific lineage differentiation for tissue regeneration [39]. It appears that adult stem cells favorably repair the tissue where they originated from. The heterogeneity (*i.e.*, proteonomic [40], transcriptonomic [41], epigenomic [42]) and secretomes [43] of adult stem cells from different tissues may account for the lineage-specific differentiation along with their resident tissue. Recent studies have shown that tissue-specific stem cells retained superiority in lineage-specific differentiation, along with their resident tissue origins and natural roles [43]. For example, adipose-derived stem cells (ADSCs) have a significant upregulation of adipogenic genes while bone marrow-derived stem cells (BMSCs) express higher magnitude of osteogenic genes after *in vitro* induction [44].

## 3. Optimal Cell Source for Cell Therapy

### 3.1. Combinations of Somatic and Stem Cells

Cell–cell interactions are important roles in cell proliferation and differentiation of MSCs. Combinations of annulus fibrosus cells with BMSCs enhanced somatic cell proliferation and extracellular matrix synthesis [45]. When stem cells were co-implanted with somatic functional cells, cell number of both cell types increased and promoted tissue regeneration *in vivo* [46].

### 3.2. Primary Cultured Cells vs. Cell Lines

As grafted cell sources, primary cultured autologous or allograft cells as the graft sources are commonly used for tissue repair because their biologic characteristics are stable. However, with primary cultured cells, the number of cell passages is finite. In contrast, immortalized cell lines can generate a large quantity of cells via many passages. However, the cell lines are rarely used in tissue regeneration research because of the high risk of tumor formation. In addition, cell lines usually lose their initial cell morphology and differentiation capacity with increasing passages, causing weak regeneration capability after cells are implanted *in vivo* [47] and abnormal alterations of cell DNA, RNA, and proteins over time during long-term culture [48].

### 3.3. Passages of Stem Cells Used for Implantation

One report indicated no significant differences in differentiation into osteogenic, adipogenic and chondrogenic tissue among tonsil-derived MSCs from passages 2 to 15, with proliferative ability decreasing after passage 15 [49]. In another report, human umbilical cord-derived MSCs in passage 30 could still affect hematopoiesis [50]. However, other studies demonstrated that favorable passage of stem cells in chondrogenic differentiation is at passage 4, which develops potential of cartilage-like tissue in MSCs [51]. In long-term passage culture studies, BMSCs decreased bone formation and increased osteogenic disorders at passage 12 [52]. Therefore, no more than 5 passages of MSCs appear to be optimal for cell growth, paracrine effects, differentiation capacity, and DNA stability in cultures [48].

### 3.4. Non-Induced Differentiation of Stem Cells vs. Induced Differentiation of Stem Cells in Tissue Repair

It often takes over several weeks to culture and induce stem cells *in vitro*, *i.e.*, 4–6 weeks for cell culture and expanding to gain enough cells for implantation, and then 2–4 weeks to induce cell differentiation at the terminal stages. Such cell manipulation remarkably decreases cell viability and regeneration capacity after these cells are implanted *in vivo* [53]. Thus, for *in vivo* studies, it seems more advantageous to use non-induced stem cells than induced stem cells (see Table 2).

In the last decade, Takahashi and Yamanaka first reprogrammed induced pluripotent stem cells (iPSCs) from somatic cells using different transcription factors [54]. The main characteristic of iPSCs have pluripotency and can replace the use of embryonic stem cells (ESC) because they possess the ability to self-renew and differentiate to three germ layers and all cell types [55]. Thus, use of iPSCs avoids ethical issues concerning the depletion of embryos in research and clinics consequently [54]. iPSCs provide great opportunities for regenerative medicine, disease modeling, and drug discovery [55]. However, several major disadvantages obstruct the application, namely, genomic modification, teratogenicity, transmission of nonhuman pathogens to humans, and cost and labor of reprogramming process [56]. Although recent advances in reprogramming footprint-free and xeno-free iPSCs made iPSCs become the most appealing but it is necessary to significantly improve reprogramming technology to overcome the limitations.

## 4. Controlled-Release Exogenous Growth Factors

One of the effective and controllable methods to apply growth factors that can augment stem cell treatments is genetic manipulation of the cells overexpressing diverse growth factors before implantation [57,58]. The genes are often introduced into the cells via non-viral or viral techniques; the former approaches are safer, but they have low transfection efficiency and typically do not result in integration of the transgene. Common viral vectors used in the previous gene therapy studies are retroviruses such as Moloney murine leukemia virus, a serotype 5 adenovirus, adeno-associated viruses, or lentiviruses [59]. Using stem cells from fat or urine transfected with vascular endothelial growth factor (VEGF) to deliver bioactive angiogenic growth factors caused endothelial cell migration and proliferation *in vivo* [60], and promoted endothelial and smooth muscle cell function recovery, increased processing of oxidation within cavernous tissue, and improved erectile dysfunction in a rat model of diabetic erectile dysfunct [61]. In addition, adult neural stem cells infected with bicistronic lentiviral vector Lv.IL-10, encoding both inerleukin-10 and green fluorescent protein GFP driven by a cytomegalovirus promoter to express interleukin-10 enhanced immune suppression, remyelination, and neuronal repair [62]. However, the long-term safety of doses of released growth factors and the risk of tumor-genesis by genetically modified stem cells with viral transfection are concerns [63].

Most growth factors have half-lives within minutes, emphasizing the importance of controlled, continuous release within a protective delivery vehicle [64]. Direct injections of growth factors were less effective in promoting tissue healing due to their rapid dilution and short half-lives. These bioactive proteins were released homogeneously from the microspheres providing stem cell niches [65]. A key advantage of microspheres is the potential for minimally invasive local delivery, allowing for optimal construct-tissue integration [66]. Bioactive microspheres loaded with therapeutic growth factors provide controlled and sustained delivery to induce cell differentiation and stimulate tissue regeneration [67].

Microspheres derived from materials such as chitosan [68], alginate [69], lactic-co-glycolic acid (PLGA) [70], and chondroitin sulfate [71] have been used as carriers for coordinated release of growth factors. Co-encapsulation of microspheres with photopolymerizable *N*-methacrylate glycol chitosan enhanced stem cell chondrogenesis *in vitro* [65] when a hydrogel matrix loaded with bone morphogenetic protein 6 and transforming growth factor-β3 was co-administered with ADSCs. Urine-derived stem cells (USCs) combined with alginate micro-beans containing three groups of growth factors (insulin-like growth factor (IGF)-1 and hepatocyte growth factor (HGF) for muscle, VEGF and fibroblast growth factor (FGF)-1 for angiogenesis, and nerve growth factor (NGF) and IGF-1 for innervation) are superior in myogenesis, revascularization, and neurogenesis to those with one group of growth factors alone when subcutaneously implanted *in vivo*. In addition, PLGA microspheres in a hyaluronic acid scaffold promoted proliferation and migration of neural stem cells via release of brain-derived neurotrophic factors and VEGF. This approach also enhanced endothelial cell attachment on biomaterials *in vitro*, all of which could be beneficial in treating central nervous system injuries [70]. A stem cell transplantation system that contained autologous ADSCs and controlled-release NGF encapsulated within PLGA microspheres enhanced therapeutic efficacy of ADSCs delivered by periurethral co-injection in a rat model of stress urinary incontinence [72].

Several natural polymers employed as biologic scaffolds carry stem cells for tissue repair, including alginate, collagen, fibrin, albumin, hyaluronan, platelet-rich plasma, and gelatin [73]. Growth factors released from the scaffolds could significantly prompt stem cell growth and differentiation, but most of these proteins cannot bind with scaffolds and so require a bridge to covalently crosslink scaffolds on one end and bind growth factors on other end. Heparin, one such element, has high levels of sulfated anionic glycosaminoglycans that contain a growth factor binding domain [74] which allows heparin to bind growth factors with high affinity while retaining its biological activity [75]. Using heparin with growth factors controls their release keeps stem cells viable after transplantation [75]. A heparin-presenting injectable nano-fiber network bound and delivered paracrine factors that mimicked stem cell paracrine effects and promoted tissue healing in ischemic tissues [76]. In addition, transforming growth factor-β1 (TGF-β1) as a chondrogenic growth factor was coupled with a heparin domain to obtain TGF-β1-conjugated F127 hydrogel, which induced chondrogenic differentiation of ADSCs *in vivo* [77].

## 5. Preconditioning Stem Cells

Preconditioning strategies in stem cell therapy increase cell survival rate and differentiation potential and enhance paracrine effects in suppressing inflammatory factors, immune responses, and fibrosis; these effects promote organ and tissue regeneration and functional recovery after cell implantation [78]. Generally, the harsh post-ischemic environment of injured tissue is associated with oxidative stress, chronic inflammation, fibrosis, extracellular matrix degradation, and immune rejection [79,80], which mediate apoptosis, poor cell survival, or low retention rate of the grafted cells [81]. Hence, prolonging stem cell survival and improving their therapeutic effects on regeneration in damaged tissues rely on prevention of anoikis, resistance against nutrient deprivation, elimination of immune rejection, and enhancement of anti-oxygen capability in the ischemic area. Three strategies are frequently involved in cell preconditioning: hypoxia, growth factors, and conditioned medium from functional cells or serum-free medium.

Hypoxia preconditioning pre-exposes cells to severe hypoxia to activate cellular defenses [81]. Hypoxia treatments vary from under 2%–5% oxygen and have enhanced cell viability [81] and proliferation of human MSCs [82]. In addition, hypoxia-preconditioned MSCs promoted angiogenesis and neurogenesis, and stimulated differentiation [83]. However, both adipogenic differentiation and chondrogenesis were inhibited when cells were exposed to hypoxia during differentiation [82].

Preconditioning of stem cells with growth factors like IGF-1 and VEGF enhance cytoprotective effects, resulting in improved efficiency of cell therapy [84,85]. IGF-1 is involved in the survival pathway [86] and has been widely used to enhance stem cell treatments [87,88]. In acute kidney injury models, preconditioning of MSCs with IGF-1 before infusion markedly improved cell migration, ameliorating renal structure damage and restoring normal renal function [88]. Similarly, cells with VEGF preconditioning also showed significant enhancement of cell proliferation, minimization of senescence, and better cell survival in a diabetes model [87]. Combining IGF-1 and FGF-2 better protected MSCs than VEGF alone [87]. MSCs-alginate constructs treated *in vitro* with TGF-1 for over 2 weeks [89] and transplanted under the dorsal skin of nude mice, generated new cartilage at 8 weeks after transplantation [89]. Before transplantation, 1 week of *in vitro* exposure to TGF-1 promotes new cartilage formation of human MSCs in alginate.

Conditioned medium from healthy functional cells can improve proliferation of multiple stem cell sources under varying structural environments [90]. MSCs isolated from diabetic animals enhanced survival, proliferation, angiogenic ability, and function of a diabetic heart when preconditioned with medium from healthy cardiomyocytes exposed to oxidative stress and high glucose [91]. VEGF levels increased in conditioned medium from neonatal rat cardiac myocytes treated with this approach, due to up-regulation of VEGF as a consequence of hypoxia [92]. Using a chondrogenic medium to precondition ADSCs effectively increased chondrogenic factor secretion (TGF-β2, TGF-β3, and IGF-1) and decreased angiogenic factor production (VEGF-A and FGF-2) [93] suggesting that conditioned medium from normal functional cells can guide the differentiation of stem cells. *In vitro* serum starvation (with or without VEGF) appears to favor cardioprotection, extracellular matrix remodeling and blood vessel maturation, all relevant for the late maturation phase in infarct healing [94].

## 6. Routes of Cell Administration

MSCs improve tissue repair mainly via differentiation and paracrine effects. Depending on therapeutic purposes, stem cells can be implanted *in vivo* via either systemic or local approaches (see Table 3). Systemic administration, including intravenous (*iv*), intraperitoneal (*ip*), and intraventricular injection, appears to mimic the route of endogenous MSCs via the circulatory system with final homing to the target sites. Local administration is designed to increase the number of implanted stem cells to the target sites for further cell fusion with the host cells or differentiation into the functional cells.

### 6.1. Systemic Administration of Stem Cells

Intravenous delivery of MSCs is the most common approach. Stem cells enter the circular system to secrete trophic factors, however a few of the cells can home to the injured site or wound tissue. Repeated intravenous administration of ADSCs reduced diabetic kidney damage in rats even at the progressive stage, and promote podocyte recovery via secretion of glial cell line-derived neurotrophic factor [103]. However, graft cells may be limited by donor cell entrapment in the lungs or destroyed in the spleen, lymph nodes, or liver after intravenous injection, which reduces the lifespan of the grafted cells after intravenous administration.

Intra-ventricular injection via interventional technology, although more invasive, is a more efficient method of stem cell delivery to the target sites. In rodent models, <1% of intravenously delivered implanted cells arrived at the target organ, compared to 10% of cells via intra-ventricular injection [100].

Intra-arterial injection significantly reduces trapping of cells in the lungs and increases the likelihood that injected cells reach the target organs in a large animal. Highly-selected intra-arterial injection can prevent the implanted cells widely distributed in other organ [104]. Intra-arterial injection of stem cells into organs had better effects than intravenous injection for tissue regeneration. In a rodent model, cell number and speed of implantation are critical factors to achieve the efficiency of cell therapy. About 1 × 10^5^ MSCs administered through the renal artery homogeneously distributed within the renal tissues, which significantly improved renal function and histological structure, compared to intravenous infusion of MSCs in a rat model of acute kidney injury [104].

In cancer treatment, intra-peritoneal administration is often used to deliver stem cells *in vivo*, and it can be used to implant stem cells or proteins from their culture in experimental cell therapy approaches. For example, in a rat model of vaginal distention injury, a single intra-peritoneal injection of concentrated media conditioned by MSCs improved urethral sphincter function via increasing leak point pressure 3 weeks after injury [105]. Although intra-peritoneal injection is simple, stem cells may inadvertently be injected into intestine [106]. Intravenous administration of MSCs was superior to intra-peritoneal therapy in reducing colon inflammation in a murine model of colitis [101]. This is because cell migratory capacity and activation of immunosuppressive properties of MSCs transplanted intravenously may more efficiently enhance immunomodulatory effects and tissue repair.

### 6.2. Local Administration of Stem Cells

Local administration is the most efficient route for cell homing and immediate generation of local action via *in situ* injection or intra-organ infusion to the damaged tissue. Direct injections of stem cells have been reported to use to repair injury in solid organs and their related tissues, such as heart, brain, spinal tissue, liver, kidney, testis, penile cavernous tissue, urethral sphincter, and skeletal muscle. For example, myocardial injection of MSCs for tissue regeneration has been combined with cytokine enhancement in the border zone to create a beneficial microenvironment for cell survival [107]. In a rodent model of streptozotocin-induced diabetes, BMSCs [108] or USCs [61] significantly enhanced endothelial regeneration, increased intracavernous pressure, and restored erectile function. Furthermore, direct injection of stem cells into the testis allows implanted stem cells to penetrate through the blood-testis barrier, improving treatment for infertility [109]. Compared to intravenous injection, intramuscular injection of BMSCs with small gap neurorrhaphy better promoted peripheral nerve regeneration and improved recovery of nerve function in a rat model of peripheral nerve injury [110]. However, *in situ* administration can be overly traumatic, and its invasiveness often causes massive bleeding and secondary damage.

Collectively, each approach to cell delivery has its own advantages and limitations, so the route of cell delivery should depend on the study’s goals, size of the target organ, and animal models to be used.

## 7. Optimal Timing for Cell Therapy

The optimal time to administer stem cell therapy is unclear and likely varies among conditions. Three phases have been identified in the tissue or organ healing process: *i.e.*, injury phase (hours), repair phase (days), and remodeling phase (weeks) [111]. In a model of myocardial infarction, impaired survival of transplanted cells during the acute injury phase due to the cytotoxic environment suggests that the early inflammatory process impairs biological and functional behaviors of engrafted cells [112]. In a subsequent study, the optimal time frame for cell therapy was 4 to 7 days after acute myocardial infarction [113], indicating that the repair phase may be a more favorable time for stem cell implantation.

In studies of enhancing skeletal muscle repair in a mouse model, intramuscular injection of stem cells 4 days post-injure[114] achieved the most favorable outcomes by increasing angiogenesis and decreasing scar tissue formation after skeletal muscle contusion [115]. Intrastriatal injections 3 days after ischemic injury yielded the highest cell engraftment, *vs.* injections administration at 6 h, 24 h, 7 and 17 days. In addition, MSCs injected 10–15 min after BSMC transplantation caused graft-*versus*-host disease occurred, but were successful injected 3 and 7 days after BMSC transplantation [116]. The primary effect occurs via trophic effects rather than cell differentiation and replacement, transplantation during the repair and remodeling phases.

## 8. Optimal Number of Cells for Injection

Delivery of appropriate number of stem cells is a pivotal factor in tissue regeneration. The optimal cell concentration for transplantation varies with different organs, tissues, and animal species. Transplanting large numbers of cells intravenously in a single treatment might not achieve a better outcome because the injected cells cause blockages in the capillaries of the lungs. In addition, injection of cells (>1 × 10^6^/mL) into an artery of a specific organ, such as the renal artery, causes cell breakdown in the glomerular capillaries [117].

In various clinical trials, the effectiveness of cell-based therapy for tissue injury depends on optimal dose of stem cell administration. In patients with steroid-refractory acute graft-*versus*-host disease, neither high (8 × 10^6^ MSCs/kg) or low (2 × 10^6^ MSCs/kg) doses of cells showed significant differences in response rate or primary disease effects [118]. For patients with spinal injury, the effective dose ranges from 0.5 × 10^6^ to 5 × 10^6^ MSCs/kg body weight of the recipient [119]. Cell dose ranged from 1 × 10^2^ cells/kg up to 2.4 × 10^9^ cells in transplantation of bone marrow mononuclear cells for cardiac applications, underwent different enriching techniques such as manual processing via Ficoll gradient *vs.* automated enrichment for MNCs via Sepax and also distinct vehicles for infusion, highlighting at least some source of variability in clinical outcomes [120]. In addition, repeated infusion of MSCs at certain intervals seems to influence the outcome [115,120]. Thus, MSC treatment may be best administered as one-time injection for acute injuries and as multiple injections for chronic diseases.

## 9. Rodents *vs.* Larger Animal Models

Autologous, allogeneic, and even xenogenous MSCs have achieved therapeutic outcomes after transplanted into rodent hosts. Human ADSCs as grafted cells significantly improved different tissue or organ function and histological structures in immunocompetent rats of a serial of diseases without immunosuppressants [121]. Implanted human bone marrow CD34^−^ cells survived and differentiated up to 42 days in a rat model of intervertebral disc degeneration [122]. Immunocompromised rodents are often used to test the therapeutic effects of human stem cells as xenogenous cells, using tracking approaches, such as green (GFP) or red (mCherry) fluorescent protein. Without previous labeling, human MSCs can be tracked by quantitative PCR (qPCR) of human Alu sequences (300 bp) or histology after labeling of human-specific antigens. These human Alu sequences are highly repeated and species-specific.

Large animal models (e.g., pig, sheep, dog, and nonhuman primates) have many similarities to humans in organ size, anatomy, physiology, metabolic status, and disease occurrence mechanism with humans. However, the cost, specialized facilities, regulatory burden, and ethical issues associated with primate models make alternative large animal models such pigs and sheep more attractive. Pigs are commonly for kidney, bladder, and ureteral tissue regeneration experiments [123]. Research on porcine iPSCs in regenerative medicine has made great progress. For example, pig iPSCs directly injected into ischemic myocardium significantly improved left ventricular function and perfusion [124] (see Table 4).

## 10. Future Directions

Adult stem cells can differentiate into multiple lineages, secrete vital factors related to proliferation and immune regulation, and home to injured sites. While such cell-based therapies are highly promising, challenges remain. More studies are needed to determine the mechanisms and biological properties of stem cells from different sources to enhance their therapeutic efficacy, and ensure safety of using stem cells in humans. In addition, it is pivotal to select the optimal routes of cell administration, timing, cell number for injection, cell sources and passages, to improve cell secretion and immune regulation, which are associated to cell survival, longevity, and overall function. Eventually, stem cell therapy likely will become a means of treating many diseases that currently lack effective treatment.

## Figures and Tables

**Table 1:** Overview of key therapeutic properties of mesenchymal stem cells in different disorders.

Types of Disorders	Key Therapeutic Properties of Stem Cells
Congenital defects Trauma or injury	Pluripotency or able to differentiate into multiple cell types; Enables maintenance of an undifferentiated phenotype in multiple subcultures; Capacity for self-renewal
Immune disorders	Immunomodulatory effects or reduce local inflammation and fibrosis formation; Counteract chemotactic signals released to recruit immune cells to the site of injury; Paracrine effects, immune tolerance or blunt host immune response
Degenerated diseases	Neruoprotection; Anti-apoptosis; Anti-aging effects; Stimulate endogenous tissue regeneration potential

**Table 2:** Comparison of non-induced and induced differentiation of stem cells in tissue repair *in vivo*.

Comparison Items	Induced Differentiation of Stem Cells	Non-Induced Differentiation of Stem Cells
Differentiation status	Differentiated	Non-differentiated
*In vitro* manipulation	More cell expansion and *in vitro* differentiation	Less
Cell viability *in vivo*	Moderate	Higher
Lifespan *in vivo*	Shorter	Longer
Secretion of trophic factors	Moderate	More
Recruitment of resident cells	Moderate	More
Corporation with resident cells	Transitional stay	Cell fusion, differentiation, and stimulation
Effect on tissue repair *in vivo*	Limitation due to short cell life-span	Acceleration to guide local stem cells differentiation

**Table 3:** Routes of stem cell implantation in solid organs *in vivo*.

Comparison Items	Systemic Administration			Local Administration	
	Intravenous Injection (*iv*)	Intra-Ventricular Injection	Intra-Peritoneal Injection (*ip*)	Intra-Organ Injection	Intra-Arterial Injection
Ratio of implanted cell retention or Differentiation [95]	1%	Up to 10%	0	10%–30%	10%–20%
Trophic effect	Yes	Yes	Yes (only)	Yes	Yes
Location of implanted cell	Lungs, Spleen, Liver [95]	Blood circulation	Omentum and mesentery [96], and most organs	Targeted organ or tissue	Targeted organ
Advantages of procedures	Easy [97]	Mainly for Rodent	Easy, particularly for rodent [98]	Immediate local action [99]	Applicable only for patients or large animals
Limitations or complications	<1% of implanted cells homing to the target organ; most in the lungs and spleen [100]	10% of the cells in target organ [100]	Might inject into intestine, less effect [101]	An open surgery is need to deliver the majority of cells in the right sites [102]	Interventional therapy is required in large animals [102]

**Table 4:** Benefits and disadvantages of animal models for stem cell therapy.

Comparison Items	Small Animals	Large Animals
Commonly used animals [125]	Mouse, rat, rabbit	Dog, pig, nonhuman primates
Commonly used cell sources	Xenogenous or allogeneic stem cells	Autologous stem cells
Optimal cell doses	0.5–2 millions/injection	1–5 millions/injection
Route of cell administration	Intravenous, intraperitoneal, intra-organ	Intra-arterial, intraperitoneal, intravenous, intra-organ
Advantages	Immunocompromised rodents (*i.e.*, NOD/SCID mice, or NOD-Rag mice) used to test human cells; Rapid tissue repair process; Used for proof-of-concept studies [125]	Most organs are similar in anatomy and physiology to humans; Used for pre-clinical studies [126]
Disadvantages [125,126]	Not optimal for ureteral, bladder, or urethral reconstruction	Ethical issues in using some models More expensive for maintaining
